# Theoretical Prediction of the Complex P-Glycoprotein Substrate Efflux Based on the Novel Hierarchical Support Vector Regression Scheme

**DOI:** 10.3390/molecules23071820

**Published:** 2018-07-22

**Authors:** Chun Chen, Ming-Han Lee, Ching-Feng Weng, Max K. Leong

**Affiliations:** 1 Department of Chemistry, National Dong Hwa University, Shoufeng, Hualien 97401, Taiwan; 610212011@ems.ndhu.edu.tw (C.C.); 610512018@gms.ndhu.edu.tw (M.-H.L.); 2 Department of Life Science and Institute of Biotechnology, National Dong Hwa University, Shoufeng, Hualien 97401, Taiwan; cfweng@gms.ndhu.edu.tw

**Keywords:** P-glycoprotein, efflux ratio, in silico, machine learning, hierarchical support vector regression, absorption, distribution, metabolism, excretion, toxicity

## Abstract

P-glycoprotein (P-gp), a membrane-bound transporter, can eliminate xenobiotics by transporting them out of the cells or blood–brain barrier (BBB) at the expense of ATP hydrolysis. Thus, P-gp mediated efflux plays a pivotal role in altering the absorption and disposition of a wide range of substrates. Nevertheless, the mechanism of P-gp substrate efflux is rather complex since it can take place through active transport and passive permeability in addition to multiple P-gp substrate binding sites. A nonlinear quantitative structure–activity relationship (QSAR) model was developed in this study using the novel machine learning-based hierarchical support vector regression (HSVR) scheme to explore the perplexing relationships between descriptors and efflux ratio. The predictions by HSVR were found to be in good agreement with the observed values for the molecules in the training set (*n* = 50, *r*^2^ = 0.96, $q_{\mathrm{CV}}^{2}$ = 0.94, RMSE = 0.10, *s* = 0.10) and test set (*n* = 13, *q*^2^ = 0.80–0.87, RMSE = 0.21, *s* = 0.22). When subjected to a variety of statistical validations, the developed HSVR model consistently met the most stringent criteria. A mock test also asserted the predictivity of HSVR. Consequently, this HSVR model can be adopted to facilitate drug discovery and development.

## 1. Introduction

Permeability glycoprotein also known as P-glycoprotein (P-gp), which belongs to the ATP-binding cassette (ABC) superfamily of transporters, can actively transport a wide range of structurally and mechanistically diverse endogenous and xenobiotic chemical agents across the cell membrane at the energy expense of ATP hydrolysis [1]. P-gp, a 170-kDa plasma membrane protein encoded by the multidrug resistance gene (*MDR1*/*ABCB1*), is expressed at high levels in various tissues such as blood–brain-barriers (BBB), gastrointestinal tract (GIT), liver, kidney, and placenta [2,3,4,5,6]. In addition, P-gp plays significant roles in cell and tissue detoxification and elimination of harmful substances per se [1]. For example, the accumulation of neurotoxic amyloid-*β* (A*β*) peptides in the brain represents a pathogenic hallmark of Alzheimer’s disease (AD), which is the most common form of dementia in aging populations [7]. It has been found that the decreased clearance rather than production of A*β* is the primary formation of the deleterious A*β* plaques in the brain [8]. The decreased elimination of A*β* from the brain into the blood can be partially attributed to the dysfunction of P-gp function, leading to the progression of AD [9,10,11]. Furthermore, it has been shown that A*β* can downregulate the P-gp expression along with other transporters and consequently lead to further accelerated neurodegeneration [12]. Hence, it has been suggested to increase A*β* clearance from the brain by restoring P-gp function of BBB to reduce A*β* brain accumulation as a new strategy in the medical treatment of the early stages of AD [13,14].

Additionally, P-gp efflux can profoundly implicate the role of drug absorption, distribution, metabolism, excretion, and toxicity (ADME/Tox) [15] that can clinically alter the administrated drug efficacy or even lead to various adverse side-effects due to drug–drug interaction (DDI) in the case of polypharmacy [16]. For instance, rifampin can interact with the P-gp substrate digoxin, leading to a lower accumulation of digoxin, as demonstrated by a clinical study [17]. Moreover, it is of particular interest to observe the subtle role played by P-gp in the central nervous system (CNS) since P-gp can affect the BBB penetration and pharmacological activities of administrated drugs [18]. The CNS-related side-effects of non-CNS drugs can be eliminated by P-gp because of their limited BBB penetration [19,20]. For instance, the P-gp substrate loperamide, which is a long-acting anti-diarrheal agent by agonizing the μ-opioid receptor, does not cause any CNS side-effects when administrated alone due to the blockage of the BBB penetration by P-gp [21]. When co-administrated with the P-gp inhibitor quinidine, loperamide produces adverse respiratory depression without significant alteration of the plasma accumulation due to its central opioid effect [22]. Conversely, P-gp can restrict or even eliminate the entry of CNS-targeted drugs into the brain, resulting in the reduction of the clinical efficacy [23].

In addition to normal tissues and organs, various types of tumor can over-express P-gp, producing multidrug resistance (MDR) [24], in which a single drug causes a non-drug resistant cell or cell line to become cross-resistant to other pharmacologically unrelated drugs due to the increase of administrated drug efflux and the decrease of intracellular drug accumulation [25]. As a result, P-gp efflux remains a major obstacle in the success of various kinds of cancer treatment [26] as well as infectious diseases [3,27]. For instance, brain tumor is one of the leading forms of malignancy and one of highest causes of cancer-related mortality among young adults aged less than 40 years and children [28] and glioma is the most common type of primary brain cancer with limited survival time and rate [29]. The CNS penetration of cediranib, which is a tyrosine kinase inhibitor for the treatment of glioma, is severely limited by the P-gp active efflux [30]. Co-administration of P-gp inhibitors is conceptually plausible and yet infeasible to circumvent MDR because of ineffective P-gp inhibitors in practical clinical applications [31,32]. Alternatively, P-gp can be considered as an anti-target in pharmaceutical research [33] especially in the field of CNS-targeted therapeutics [34,35]. Nevertheless, not all of marketed drugs have to be P-gp non-substrates provided that their therapeutic index is large with respect to the P-gp efflux ratio (ER) [36,37]. For instance, risperidone and 9-hydroxyl risperidone are clinically approved therapeutic agents for the treatment of schizophrenia even though they are P-gp substrates [38]. Accordingly, it is conceivable to expect that quantitative measure, viz. P-gp substrate efflux ratio, is more clinically relevant than qualitative classification, viz. substrate/non-substrate classification.

Of various in vitro assays to measure the efflux ratio [39,40,41,42], the monolayer efflux assay is the most relevant to drug distribution and the most commonly used in practice [20], in which the polarized epithelial cells, such as Madin–Darby canine kidney (MDCK) cells, are transfected with the *MDR1* gene, followed by measuring the ratios between basolateral-to-apical (B→A) apparent permeability (*P*_app_) and apical-to-basolateral (A→B) *P*_app_ [43].

(1) $$\mathrm{ER}=\frac{P_{\mathrm{app}}\left(B\to A\right)}{P_{\mathrm{app}}\left(A\to B\right)},$$

(2) $$P_{\mathrm{app}}=\frac{1}{AC_{0}}\cdot \frac{dQ}{dt},$$

where *P*_app_ is evaluated using the membrane surface area (*A*), initial dosing concentration of the test molecule (*C_0_*) in the donor compartment, and the amount of molecule transported per time (*dQ*/*dt*) in the receiver compartment [44]. Normally, molecules with ER > 2 are classified as P-gp substrates [39].

In contrast to in vitro and in vivo assays, in silico approaches are usually swift, inexpensive, less labor intensive, and less time-consuming for drug discovery and ADME/Tox profiling [45,46]. In fact, numerous P-gp classification structure–activity relationship (CSAR) models have been published elsewhere [47,48,49,50,51,52,53,54,55,56,57,58,59,60,61,62,63,64,65,66,67,68], whereas in silico quantitative studies of efflux ratio are scant [69,70,71]. Nevertheless, it is highly challenging to accurately model P-gp–substrate interactions [72] since P-gp is highly promiscuous per se as the result of the fact that P-gp can undergo substantial conformational changes upon binding with various ligands as illustrated by Figure 1 of Leong et al. [73]. In addition, P-gp has multiple substrate binding sites, as reported [72,74,75,76,77]. The mechanism of P-gp substrate efflux is far more complicated than P-gp–substrate interactions since P-gp substrate efflux can take place through various routes in that substrates can be actively transported by P-gp from the cytoplasm into the extracellular environment in an energy-dependent manner or through a protein channel positioned between the inner and outer leaflets of the lipid membrane, as illustrated by Figure 2 of Edwards [78]. In addition to active transport, P-gp substrates can also passively diffuse from the cytoplasm into the extracellular environment through transcellular diffusion and/or paracellular route, as illustrated by Figure 1 of Balimane et al. [79]. Notably, the P-gp substrate vinblastine, for instance, can be both passively diffused and actively transported [80]. As such, those modeling schemes employed by previously published investigations can only render the direct protein-ligand interactions and they are not suitable to model the efflux ratio. Conversely, quantitative structure–activity relationship (QSAR) schemes, which are a mathematic means to establish the relationship between biological activity and chemical characteristics, provide better approaches to model the efflux ratio since they can take into account any mechanisms that can occur through complex routes [81].

The complexity of P-gp mediated efflux can be problematic once the delicate roles played by those associated chemical features, viz. descriptors in QSAR models, are considered. For instance, inhibitors, modulators, and substrates can interact with P-gp using the hydrophobicity, hydrogen-bond acceptor (HBA), and hydrogen-bond donor (HBD) features [47,73,82]. Accordingly, hydrophobicity, HBA, and HBD can simultaneously enhance and reduce the P-gp efflux, and it is plausible to expect extremely nonlinear relationships between those chemical features and efflux ratio, suggesting that those linear models can yield significant prediction errors once applied to the test samples that are very different from their training patterns.

Thus, it seems extremely difficult, if not completely impossible, to develop a sound in silico model to predict the P-gp substrate efflux ratio to compressively take into account those critical factors mentioned above. A solution to such challenge, however, can be obtained by the novel hierarchical support vector regression (HSVR) scheme proposed by Leong et al. [83] because HSVR can render the complex and varied dependencies of descriptors. As such, HSVR can simultaneously possess the advantageous characteristics of a local model and a global model, viz. broader coverage of applicability domain and higher level of predictivity, respectively. Furthermore, HSVR is designated to circumvent the “mesa effect” [84] in that the performance of a developed model deteriorates dramatically when applied to extrapolated predictions as demonstrated elsewhere [85,86]. In other words, HSVR is insensitive to outliers as compared with the other predictive models that is of critical importance to a predictive model [87]. Herein, the objective of this investigation was to develop an accurate, fast, and predictive in silico model based on the HSVR scheme to predict the P-gp substrate efflux ratio to facilitate drug discovery to design molecules with a preferable ADME/Tox profile.

## 2. Results

### 2.1. Data Compilation

More than 550 compounds were collected after comprehensive literature search. data curation was carefully carried out by eliminating those compounds: (i) with only qualitative array results (i.e., substrate or non-substrate); (ii) without specific ER values; or (iii) chemical structures. In addition, cells used to express P-gp protein also play a significant role in determining ER values. For instance, the measured ER values of astemizole were 2.16 and 0.6 assayed in MDCK and human colon adenocarcinoma (Caco-2) cells, respectively [51,88]. Of various assayed cells, 63 molecules tested in MDCK cells were selected from various sources [23,39,88,89,90,91,92,93,94,95,96,97,98,99,100,101] since it constituted the largest amount of data. The data size is seemingly small since several CSAR models have been derived based on rather large amounts of data. For instance, Li et al. [66] built various predictive models based on 423 P-gp substrates and 399 non-substrates compiled from numerous sources. Nevertheless, their data were generated from different assay conditions (e.g. different cell lines), leading to high levels of data heterogeneity. QSAR models, conversely, are vulnerable to data inhomogeneity [102]. Additionally, some molecules such as selenium-containing ones [103] were excluded because their topological descriptors, for instance, cannot be enumerated. Those ER values were discarded when they were not consistent with their measured *P*_app_ (B→A) and *P*_app_ (A→B) values [104]. Recently, the efflux ratios of more than 4000 Amgen in-house compounds were measured [105]. It is plausible to expect that the great sample amount and data consistency can furnish a good ER pool. Unfortunately, those chemical structures are proprietary, leading to the fact that there are only limited quantitative data with chemical structures available in the public domain to date. Those factors partially contribute to the fact that there is no genuine QSAR model has been published.

As such, only very limited data samples with available chemical structures and consistent assay conditions were recruited in this study to maximize the structural diversity and to maintain data homogeneity after purging inappropriate data based on above-mentioned criteria. Table S1 lists the SMILES strings, CAS registry numbers, efflux ratio values, and literature references of all molecules collected in this study.

### 2.2. Data Partition

Of all molecules adopted in this study, 50 and 13 molecules were randomly assigned to the training set and test set, respectively, with a ca. 4:1 ratio as suggested [106]. Figure S1 displays the projection of all molecules enrolled in this investigation in chemical space, spanned by the first three principal components (PCs), explaining 94.6% of the variance in the original data. As illustrated, both datasets exhibited high levels of similarity in the chemical space. Furthermore, the high levels of biological and chemical similarity between both datasets can also be validated by Figure S2, which shows the histograms of log ER, molecular weight (MW), polar surface area (PSA), number of HBA, and number of HBD in density form for all molecules in the training set and test set. Thus, it can be asserted that there was no substantial bias in datasets.

### 2.3. SVRE

**Figure 1:**
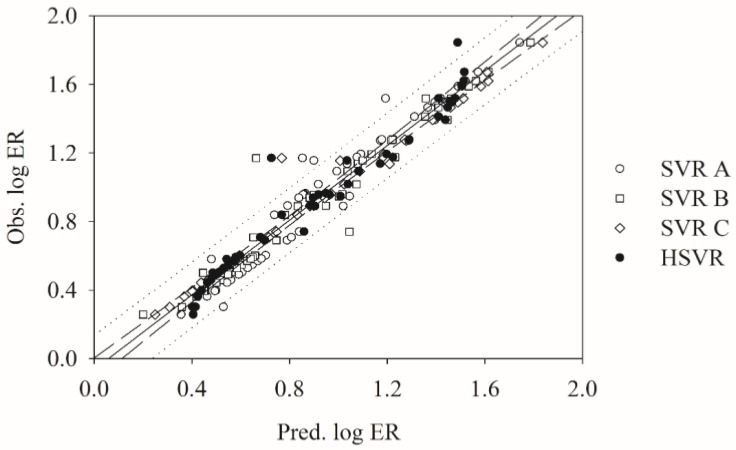
Observed log ER vs. the log ER predicted by SVR A (open circle), SVR B (open square), SVR C (open diamond), and HSVR (solid circle) for the molecules in the training set. The solid line, dashed line, and dotted lines correspond to the HSVR regression of the data, 95% confidence.

**Figure 2:**
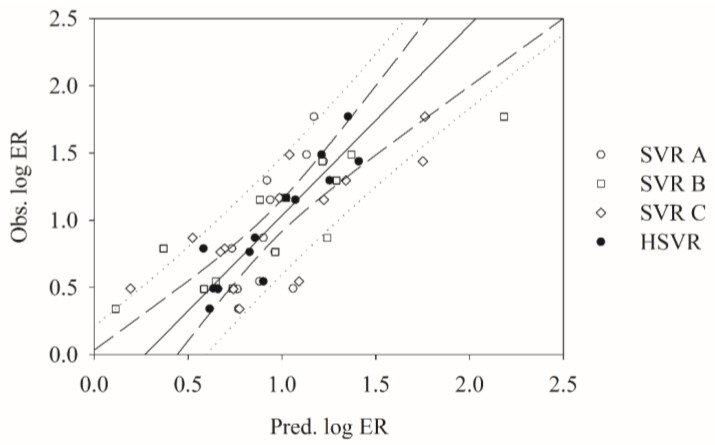
Observed log ER vs. the log ER predicted by SVR A (open circle), SVR B (open square), SVR C (open diamond), and HSVR (solid circle) for the molecules in the test set. The solid line, dashed line, and dotted lines correspond to the HSVR regression of the data, 95% confidence interval for the HSVR regression, and 95% confidence interval for the prediction, respectively.

Of all generated SVR models using various combinations of descriptors and runtime parameters, three SVR models, denoted by SVR A, SVR B, and SVR C, were assembled to construct the SVR ensemble, which was further subjected to regression by another SVR to generate the HSVR model. Table S2 summarizes the optimal runtime parameters of SVR A, SVR B, and SVR C. These three SVR models, which adopted 4, 6, and 3 descriptors (Table 1), respectively, were selected based on their individual performances on all molecules and statistical analyses in the training set and test set. Table S1 lists the predicted log ER values. Table 2 and Table 3 summarize the associated statistical analyses of these three SVR models in the training set and test set, respectively. Figure 1 and Figure 2 display the scatter plots of observed versus the predicted log ER values by SVR A, SVR B, and SVR C for the molecules in the training set and test set, respectively.

Figure 1 shows that the predictions by SVR A, SVR B, and SVR C are in good agreement with the observed values for most of the molecules in the training set as further manifested by their small RMSDs, average deviations, standard deviations (*s*), and larger *r*^2^ parameters (Table 2). Of 50 training samples, SVR A, SVR B, and SVR C gave rise to 28, 3, and 2 predictions, which deviated from the experimental values by more than 0.10, respectively. It can be further observed in Figure 1 that most of the points predicted by SVR C generally lie on or are closer to the regression line when compared with SVR A and SVR B. As a result, SVR C produced the lowest MAE (0.02), *s* (0.06), and RMSE (0.06) and the highest *r*^2^ parameter (0.98), suggesting that SVR C performed better than SVR A and SVR B for the molecules in the training set. Nevertheless, the predictions of quinidine (**48**) by SVR A, SVR B and SVR C unanimously yielded the maximum residuals of 0.32, 0.51 and 0.40, respectively, denoting that SVR A executed better than SVR B and SVR C.

The predictions by SVR A, SVR B, and SVR C in the test set are also in good agreement with the experimental values (Figure 2). Nevertheless, most of the residuals obtained by the three SVR models in the test set are more than 0.15 (11, 11, and 8, respectively). It can be further observed in Table 3 that the mean absolute errors computed by SVR A, SVR B, and SVR C unequivocally increase from 0.11, 0.07, and 0.02 in the training set to 0.29, 0.22, and 0.24 in test set, respectively. The other statistical parameters also suggest that the performances of these three models in the SVRE slightly decline from the training set to the test set (Table 2 and Table 3). The maximum residual computed by SVR C in the test set was yielded from the prediction of cimetidine (**13**) with an absolute residual of 0.55, which were only 0.34 and 0.10 by SVR A and SVR B, respectively. Similarly, vinblastine (**58**) was best predicted by SVR C with an absolute residual of 0.01, whereas SVR A and SVR B gave rise to absolute errors of 0.60 and 0.41, respectively.

Furthermore, SVR A, SVR B, and SVR C yielded the *q*^2^ values of 0.54, 0.75, and 0.60 in the test and the cross-validation correlation coefficients $q_{\mathrm{CV}}^{2}$ of 0.01, 0.01, and 0.07 in the training set, respectively (Table 2 and Table 3). When subjected to the *Y*-scrambling test, SVR A, SVR B, and SVR C gave rise to the $\langle r_{s}^{2}\rangle$ values of 0.02, 0.03, and 0.03, respectively (Table 1). The almost zero values of $\langle r_{s}^{2}\rangle$ as well as substantial differences between corresponding *r*^2^ and $\langle r_{s}^{2}\rangle$ signify that those three SVR models in the ensemble are not the result of chance correlation [107]. Conversely, the substantial differences between *r*^2^ and *q*^2^ and between *r*^2^ and $q_{\mathrm{CV}}^{2}$ imply the over-fitting characteristics of these three models that actually can be further manifested by their small $q_{F1}^{2}$, $q_{F2}^{2}$, $q_{F3}^{2}$, and *CCC* values (Table 3). As a result, it is plausible to expect that these models are local models per se, which have limited coverage of applicability domain (vide infra) [108].

### 2.4. HSVR

The HSVR model was produced by the regression of the SVR ensemble based on the predictions of all molecules and statistical evaluations in the training set (Table S1 and Table 2). Table S2 lists the optimal runtime conditions for the final SVR model. It can be observed in Figure 1 that the HSVR model showed better prediction accuracy than SVR A, SVR B, and SVR C for the molecules in the training set because the distances between the predictions by HSVR and regression line are generally between the largest ones and smallest ones produced by its SVR counterparts in the ensemble. However, HSVR executed better than any of SVR models in the ensemble in some cases. The predictions of desloratadine (**19**) by SVR A, SVR B, SVR C, and HSVR, for instance, yielded absolute residuals of 0.10, 0.06, 0.01, and 0.00, respectively. Statistically, HSVR performed better than SVR A and SVR B, whereas SVR C, in turn, functioned negligibly better than HSVR, as manifested by those parameters listed in Table 2. For example, SVR A, SVR B, SVR C, and HSVR yielded the *r*^2^ values of 0.95, 0.95, 0.98, and 0.96, respectively.

When applied to the test samples, HSVR only showed insignificant performance decreases from the training set to the test set. For instance, RMSE increased from 0.10 in the training set to 0.21 in the test set (Table 2 and Table 3). However, the maximum residual declined from 0.45 in the training set to 0.42 in the test set. Figure 2 displays that HSVR showed better performance than SVR A, SVR B, and SVR C in the test set. The performance predominance of HSVR can be further manifested by those statistical parameters listed in Table 3. For instance, SVR A, SVR B, SVR C, and HSVR gave rise to MAE values of 0.29, 0.22, 0.24, and 0.17, respectively. Similar observation that HSVR generated smaller absolute residuals than its counterparts in the ensemble can also be found in the test set. The absolute prediction error of paliperidone (**41**), for instance, was 0.14 given rise by HSVR, whereas SVR A, SVR B, and SVR C produced residuals of 0.57, 0.25, and 0.30, respectively. When compared with its counterparts in the ensemble, HSVR generally produced consist and small errors in both training set and test set as manifested by those parameters associated with error listed in Table 2 and Table 3, suggesting that HSVR has broader coverage of applicability domain. Additionally, HSVR yielded the smallest differences between *r*^2^ and $q_{\mathrm{CV}}^{2}$(0.02) and between *r*^2^ and *q*^2^ (0.13), indicating that HSVR was a well-trained model or no over-fitting effect was observed because it will otherwise produce at least one significant difference among those parameters. Similarly, the possibility of chance correlation of HSVR can be eliminated by *Y*-scrambling since it also produced an almost zero $\langle r_{s}^{2}\rangle$ (0.03) and marked difference between *r*^2^ and $\langle r_{s}^{2}\rangle$ (Table 2) [107].

### 2.5. Predictive Evaluations

**Figure 3:**
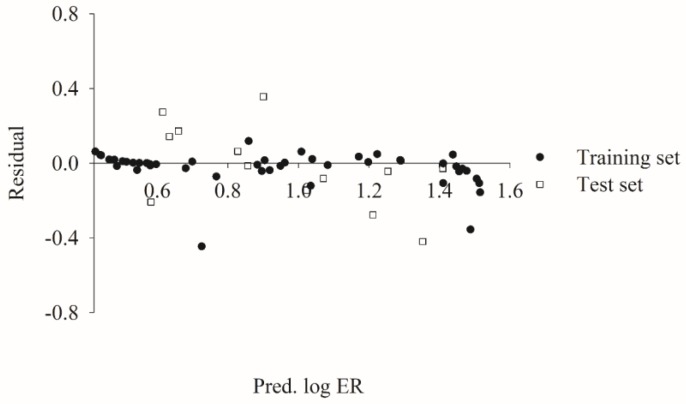
Residual vs. the log ER predicted by HSVR in the training set (solid circle) and test set (open square).

Figure 3 displays the scatter plots of the residual vs. the log ER values predicted by HSVR for the molecules in the training set and test set. It can be observed that the residuals are approximately evenly distributed on both sides of *x*-axis along the range of predicted values in both datasets, suggesting that there is no systematic error associated with the HSVR model [102]. The unbiased predictions can be further exhibited by its almost negligible average residuals that were −0.02 and −0.02 in the training set and test set, respectively (Table S1).

The predictivity of generated HSVR model was further evaluated by the validation requirements proposed by Golbraikh et al. [109], Ojha et al. [110], Roy et al. [111], and Chirico and Gramatica [112] (Equations (18)–(21)) in the training set and test set. Table 4 summarizes the results, from which it can be observed that HSVR maintained similar high levels of performance in the training set and test set. Additionally, HSVR fulfilled all validation requirements, indicating that this predictive model is highly accurate and predictive.

### 2.6. Mock Test

**Figure 4:**
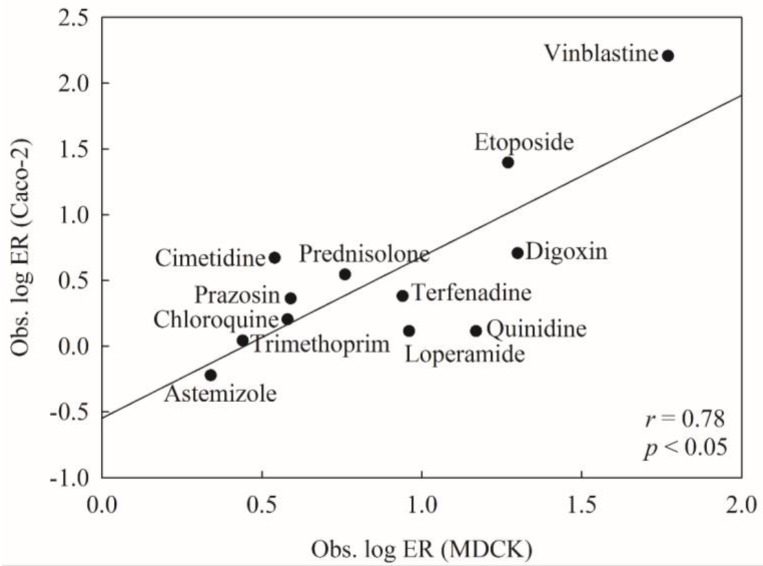
The observed log ER values (Caco-2) vs. the observed log ER values (MDCK).

To mimic real world challenges, the developed HSVR model was further tested on the P-gp substrates assayed by Crivori et al. [51]. Of all marketed drugs measured by Crivori et al., 12 were also enrolled in this study, yielding a good way to calibrate the testing system. However, these molecules were measured in Caco-2 cells, whereas all of the molecules adopted in this study were tested in MDCK cells, suggesting that those compounds assayed by Crivori et al. are not qualified as the second external or test set since those validation criteria (vide supra) are not applicable to these compounds. To eliminate the discrepancy between both assay systems, the linear correlation between both assay systems for those common molecules was first inspected and the obtained scatter plot is illustrated in Figure 4. It can be observed that the experimental values in both systems were modestly correlated with each other well with an *r* value of 0.78. Thus, it is plausible to examine the HSVR model with those novel P-gp substrates assayed in Caco-2 cells.

**Figure 5:**
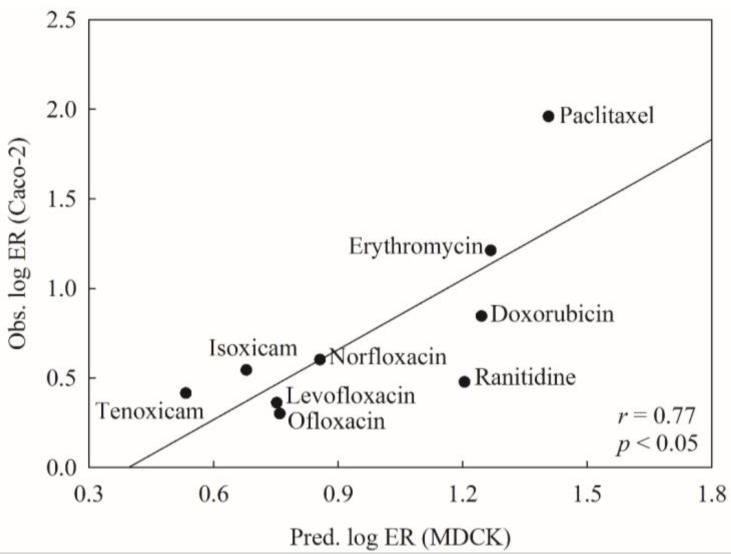
The observed log ER values (Caco-2) vs. the predicted log ER values (MDCK).

Figure 5 displays the tested results of the nine novel drugs. It can be observed that the *r* value between experimental log ER obtained in the Caco-2 cells and predicted log ER in the MDCK cells was 0.77. The negligible difference between both numbers (0.78 vs. 0.77) suggests that the predictions by the HSVR model can almost reproduce the experimental observations and this mock test unequivocally assured the predictive capability of HSVR.

## 3. Discussion

Collectively, seven descriptors were adopted in this study. Intrinsically, the sample-to-descriptor ratio was ca. 7:1, which is significantly larger than 5, viz. the minimal requirement to lessen the probability of chance correlations in a predictive model [113]. However, the process of P-gp substrate efflux is complex since it can take place thought various routes (vide supra). As such, different descriptors were adopted by different classification models. Of various descriptors selected by qualitative predictive models, hydrophobic, HBA, and HBD are the most frequently selected chemical features, as illustrated by the model proposed by Penzotti et al. [47]. However, the analysis of Amgen in-house compounds can reveal that HBD and topological PSA (tPSA) are the predominant factors associated with ER [105].

**Figure 6:**
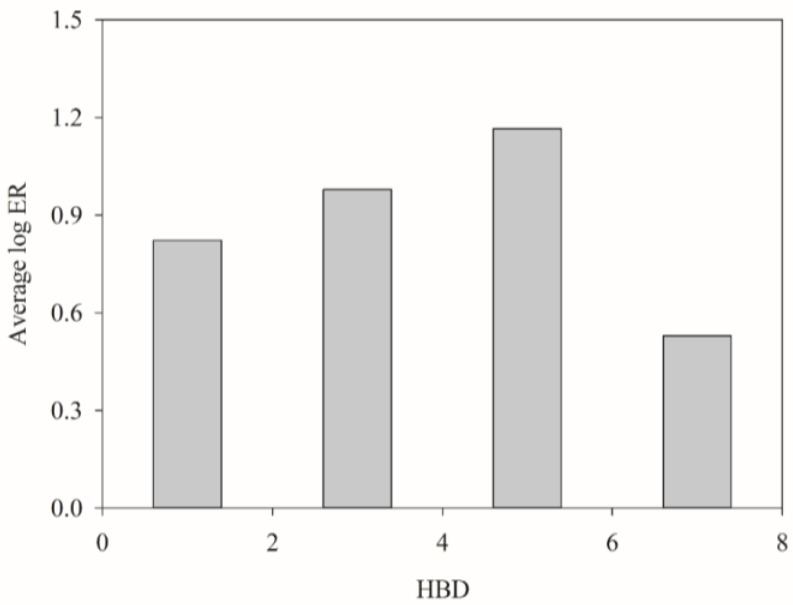
Average log ER vs. the distribution of HBD.

Figure 6 displays the average log ER for each histogram bin of HBD for all molecules selected in this study. It can be observed that the average log ER value initially increased with HBD when HBD was no more than 6 and then subsequently decreased when HBD was more than 6. Such positive dependence of log ER on HBD is, in fact, consistent with the analysis made by Hitchcock et al. [105]. However, those Amgen in-house compounds had HBD of no more than 5, leading to an only positive relationship between log ER and HBD. Such discrepancy in both systems can be conceivably attributed to the fact that the initial P-gp substrate binding can be enhanced by HBD as illustrated by the pharmacophore models of Penzotti et al. [47], whereas the consequent transport of the substrates into the extracellular environment can be hampered by too many HBDs, plausibly because of the increase in water desolvation energy [114] and the decrease in membrane fluidity [115]. As such, a nonlinear relationship between HBD and log ER was yielded consequently.

**Figure 7:**
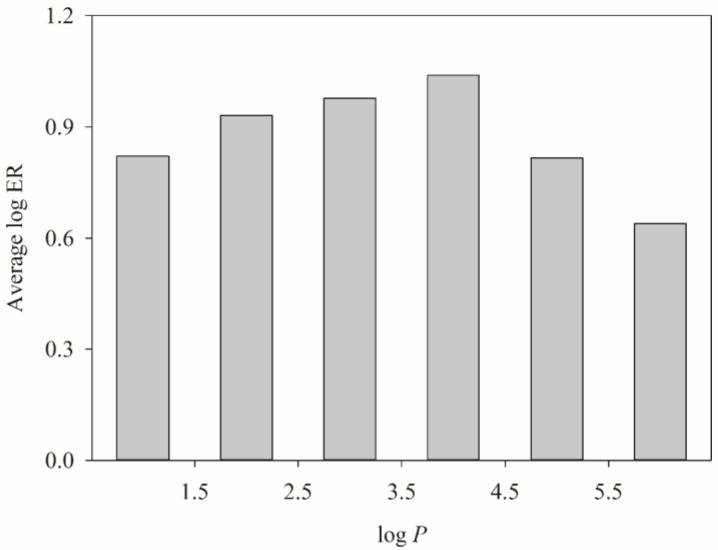
Average log ER vs. the distribution of log *P*.

It has been observed that hydrophobicity, which normally can be represented by log *P*, plays an important role in P-gp–substrate interaction due to the hydrophobic nature of the substrate binding pocket, resulting in stronger P-gp substrate binding for those more hydrophobic substrates [116]. Nevertheless, the interaction between substrates and lipid bilayer as well as the release of substrates into the extracellular environment also depend on the hydrophobicity of substrates (vide supra), leading to a nonlinear relationship between log *P* and log ER. Figure 7 displays the average log ER for histogram bin of log *P* for all molecules enlisted in this study. It can be observed that the average log ER initially increased with log *P* when log *P* was smaller and decreased with log *P* when log *P* became higher. Such observation is qualitatively similar to the trend of *P*_app_ (A→B) found by Hitchcock et al. [105].

**Figure 8:**
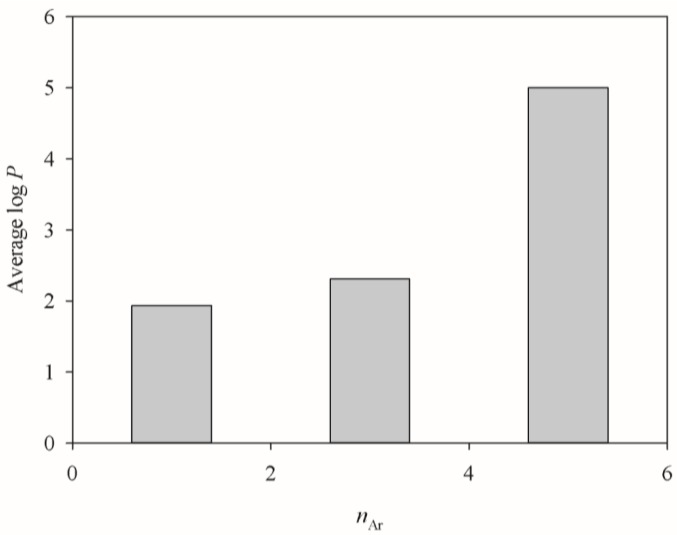
Average log *P* vs. the distribution of number of aromatic ring (*n*_Ar_).

Nevertheless, it is unusual to observe that log *P* was not included in this study, whereas the number of aromatic rings (*n*_Ar_) was enlisted in this study. Such inconsistency can be realized by the fact that the average log *P* values increased with *n*_Ar_ for all of molecules included in this study, as illustrated in Figure 8, which displays the average log *P* versus the distribution of *n*_Ar_. As such, it is plausible to replace log *P* by *n*_Ar_. Furthermore, it has been found that aromatic ring moieties are important in substrate recognition and efflux modulation [117,118]. More importantly, the empirical observation has indicated that models with the selection of *n*_Ar_ unanimously showed better performance than those with the selection of log *P* (data not shown).

**Figure 9:**
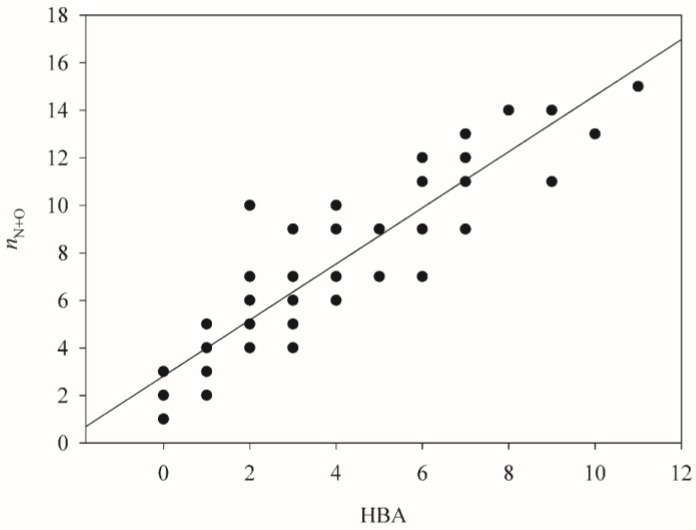
The number of nitrogen and oxygen (*n*_N+O_) vs. HBA.

The significant role of HBA in the P-gp–substrate interaction has been manifested by molecular docking simulations [71] as well as numerous qualitative models. Additionally, it has been suggested that HBA can enhance P-gp-mediated efflux [56]. Nevertheless, it is unusual to observe that none of SVR models in the ensemble has adopted HBA, plausibly because the descriptor number of nitrogen and oxygen atoms (*n*_N+O_) correlated well with HBA as demonstrated by Figure 9, which displays *n*_N+O_ versus HBA. In fact, Desai et al. [56] adopted *n*_N+O_ instead of HBA as the substrate classification criterion. Furthermore, empirical model development has shown that models with the selection of *n*_N+O_ executed better than those with the selection of HBA (data not shown). As a result, the descriptor *n*_N+O_ was selected in lieu of HBA.

The descriptor tPSA is a modified version to swiftly calculate the polar surface area only based on the additive polar surface areas [119]. The recursive partitioning (RP) model of Joung et al. [68] indicated the significant role of PSA in classifying molecules as P-gp substrates/non-substrates. Moreover, Hitchcock et al. also found the profound contribution of tPSA to P-gp mediated efflux (vide supra). Accordingly, the more sophisticated version of PSA was adopted in this study since it can function as polarity and hydrogen-bonding features [66].

It has been observed that the substrate size, which can be characterized by molecular weight (MW), molecular volume (*V*_m_), and total surface area (SA), can have a large impact on P-gp–substrate interaction as well as passive permeability [120]. Nevertheless, it has been suggested that both *V*_m_ and SA can be better metrics to estimate the actual molecular size [121], and MW, conversely, was closely associated with *V*_m_ with an *r*^2^ values of 0.98 for the molecules enlisted in this study. In fact, it has been postulated that *V*_m_ rather than MW is a better metric to associate with ER [122]. Accordingly, *V*_m_ and SA were adopted to render the size effects, whereas MW was discarded to reduce the probability of spurious correlations.

It has been found that P-gp substrates generally have more rotatable bonds than non-substrates since more flexible molecules can be more easily to adopt favorable orientation to interact with P-gp [49,66,123]. In fact, non-CNS drugs are more flexible than their CNS counterparts [23] since molecules with more conformational flexibility can favor the internal H-bond formation, which, in turn, can enhance the passive membrane permeability [124]. As such, substrate conformational flexibility, which can be characterized by the number of rotatable bond (*n*_Rot_), can facilitate not only the active transport but also passive permeability of P-gp substrates, and *n*_Rot_ was adopted in this investigation.

Gunaydin et al. [69] only took into account the contribution of the differences between free energy in water (*G*_H2_o) and that in chloroform (*G*_CHCl3_), viz. Δ*G*_H2O−CHCl3_, since it was hypothesized that P-gp undergoes a conformation change from the intercellular-facing state to extracellular-facing state upon binding with substrates. As such, the transported substrates experience from a lipophilic environment into a hydrophilic one. In addition to Δ*G*_H2O−CHCl3_, the contribution of Δ*G*_DMSO−CHCl3_ was also computed in this study to mimic the assay conditions. Nevertheless, neither of the solvation free energy differences was selected in this study due to their insignificant contribution to ERs (data not shown), plausibly because the P-gp conformation change can only account for a small part of the whole complicated efflux process and, additionally, passive permeability is not resulted from the P-gp conformation change. The predictive model of Gunaydin et al. [102], nevertheless, was derived only based on 12 marketed drugs that cannot comprehensively render the complex efflux. As such, more descriptors will be required in case of more diverse samples.

Didziapetris et al. [63] proposed the “rule-of-fours,” which states that molecules with: (i) *n*_N+O_ ≥ 8; (ii) MW > 400; and (iii) acid p*K*_a_ > 4 are likely to be P-gp substrates. Of all molecule with ER > 2 selected in this study, viz. substrates, approximately 32%, 52%, and 100% can meet the criteria *n*_N+O_ ≥ 8, MW > 400, and acid p*K*_a_ > 4, respectively, and only 29% can completely fulfill those three criteria. Actually, Li et al. [66] also found that only ca. 34% of samples can simultaneously meet those three criteria. Furthermore, it is not unusual to observe that different rules have been proposed to classify molecules into P-gp substrates/non-substrates. Desai et al. [56], for instance, have proposed the molecules with TPSA >100 Å^2^ and most basic p*K*_a_ > 8 have higher probability to be substrates. The inconsistency in various proposed rules can be plausibly explained as those rules were derived only based on linear analyses of those P-gp substrates/non-substrates. However, such bisection is not always true, as manifested by the naïve Bayesian classifiers built by Li et al. [66]. In addition, the size and hydrophobicity of substrates can affect the substrate-membrane interactions nonlinearly [125]. Further complexity can be raised once the P-gp substrate efflux is considered instead of P-gp substrate/non-substrate classification since the P-gp substrate efflux can take place through various routes (vide supra), leading to nonlinear relationships between some descriptors and log ER, such as HBD and log *P* (Figure 6 and Figure 7). Numerous attempts have been made in this study to develop various partial least square (PLS) models to accommodate the novel 2-QSAR scheme [86] and no satisfactory models were produced (data not shown). Conversely, the accurate and predictive HSVR can comprehensively describe such nonlinear dependence of log ER on descriptors.

Moreover, it has been observed that P-gp and other ABC members, namely breast cancer resistant protein (BCRP/ABCG2) and multidrug resistance-associated protein 4 (ABCC4/MRP4), play a critical role in BBB permeability [126], which can take place via various routes [127] in addition to the already complicated P-gp mediated efflux. As such, it is plausible to expect that it is extremely difficult to develop a sound in silico model to predict BBB permeability if not entirely impossible [128]. The development of an accurate in silico model in this study to predict the P-gp substrate efflux can pave the way to establish a sound theoretical model to predict the BBB permeability in the future. Most molecules adopted in this study are marketed drugs for treating various illnesses, such as HIV infection, allergy symptoms, rheumatoid arthritis, hypertension, diarrhea, and different types of cancer in addition to assorted CNS-related disorders (Supplementary Materials Table S1). The broad spectrum of therapeutic agents unequivocally indicates that the data samples are structurally diverse, which can be further manifested by the fact that the average minimum distance between two molecules, viz. the distance between two nearest neighbors, in the chemical space was 2.06 with an standard deviation of 1.39 and the maximum distance between two collected samples was 29.57 (Supplementary Materials Figure S1), giving rise to an ratio of ca. 1:14. As such, it is plausible to expect that developed HSVR should have a larger coverage of applicability domain accordingly, which is an important characteristic for a predictive model in practical application. More importantly, the derived HSVR model and published P-gp substrate/non-substrate classification models can work in a synergistic fashion, in which the latter can be used to identify those P-gp substrates and the former can be deployed to predict their efflux ratios.

## 4. Materials and Methods

### 4.1. Data Compilation

A sound predictive model can only be built based on good quality of sample data [102]. To compile quality data for this study, a comprehensive literature search was conducted to retrieve efflux ratio values from various sources to maximize the structural diversity. If there were two or more available efflux ratio data for a given compound and in close range, the average values were then taken to warrant better consistency. Further data curation was carried out by cautiously inspecting molecular structures to remove those molecules without definite stereochemistry.

### 4.2. Molecular Descriptors

All of the molecules enlisted in this study were subjected to full geometry optimization using the density functional theory (DFT) B3LYP method with the basis set 6-31G(d,p) by the *Gaussian 09* package (Gaussian, Wallingford, CT) in the dimethyl sulfoxide (DMSO) solvent system using the polarizable continuum model (PCM) [129,130] to mimic the experimental conditions. These geometries were confirmed to be real minima on the potential energy surface by force calculations when no imaginary frequency was obtained. Additionally, atomic charges were also calculated by the molecular electrostatic potential-based method of Merz and Kollman [131] and the highest occupied molecular orbital energy (*E*_HOMO_), lowest unoccupied molecular orbital energy (*E*_LUMO_), free energy (Δ*G*), and dipole (*μ*) were also retrieved from the optimization calculations since those quantum mechanics descriptors have been adopted previously. As such, it is of necessity to employ a more sophisticated quantum mechanics method to optimize those selected molecules and to calculate their associated descriptors.

The *Discovery Studio* package (BIOVIA, San Diego, CA) and *E-Dragon* (available at the web site http://www.vcclab.org/lab/edragon/) were also utilized to calculate more than 200 one-, two-, and three-dimensional molecular descriptors of those optimized molecules. These descriptors can be classified as electronic descriptors, spatial descriptors, structural descriptors, thermodynamic descriptors, topological descriptors, and E-state indices.

Data filtering was initially performed by removing those descriptors missing for at least one sample or showing little or no discrimination against all samples. Furthermore, only one descriptor should be kept among those descriptors with intercorrelation values of *r*^2^ > 0.8 to reduce the probability of spurious correlations as postulated by Topliss and Edwards [113]. It is not uncommon to observe that certain descriptors with broader ranges outweigh those with narrower ranges because of substantial variations in magnitudes. Nevertheless, such problem can be resolved when the non-descriptive descriptors, viz. real variable descriptors, are normalized with the following equation [132]

(3) $$\chi_{ij}=\left(x_{ij}-\langle x_{j}\rangle \right)/{\left[\sum_{i=1}^{n}{\left(x_{ij}-\langle x_{j}\rangle \right)}^{2}/\left(n-1\right)\right]}^{1/2}$$

where $x_{ij}$ and $\chi_{ij}$ represent the original and normalized *j*th descriptors of the *i*th compound, respectively; $\langle x_{j}\rangle$ stands for the mean value of the original *j*th descriptor; and *n* is the number of samples.

Descriptor selection plays a pivotal role in determining the performance of predictive models [133]. More descriptors will be needed once there are more training samples with more diverse structures [102]. Conversely, it is highly possible to yield an over-trained model when there are too many selected descriptors [134]. The descriptor selection was initially executed by genetic function approximation (GFA) using the QSAR module of *Discovery Studio* due to its effectiveness and efficiency [135]. Further descriptor selection was carried out by the recursive feature elimination (RFE) method, in which the predictive model was repeatedly generated by all but one of descriptors. The descriptors were then ranked according to their contributions to the predictive performance; and the descriptor with least contribution was discarded [136].

### 4.3. Data Partition

The collected molecules were divided into two datasets, namely the training set and test set, to develop and to verify the predictive models using the Kennard–Stone (KS) algorithm [137] implemented in *MATLAB* (The Mathworks, Natick, MA, USA) with an approximate 4:1 ratio as suggested [106]. It has been suggested that a sound model can be derived only based on chemically and biologically similar training samples and test samples [138]. As such, the data distribution was carefully examined to ensure the high levels of biological and chemical similarity in both datasets.

### 4.4. Hierarchical Support Vector Regression

Support vector machine (SVM) proposed by Vapnik et al. [139] was initially designated for use in classification and consequently modified for regression problems by nonlinearly mapping the input data into a higher-dimension space, in which a linear regression is performed [140]. SVM regression takes into account both the training error and the model complexity as compared with the traditional regression algorithms, which develop predictive models by minimizing the training error. As such, SVM performs better than traditional regression methods because of its advantageous characteristics, namely dimensional independence, limited number of freedom, excellent generalization capability, global optimum, and easy to implement [141].

Similar to other linear or machine learning (ML)-based QSAR techniques, SVM has to tradeoff between the characteristics of a global model, viz. broader coverage of applicability domain (AD), and a local model, viz. higher level of predictivity [108]. This seeming dilemma, nevertheless, can be plausibly resolved using the hierarchical support vector regression (HSVR) scheme, which was initially proposed by Leong et al. and was derived from SVM [83], because HSVR can simultaneously take into consideration both seemingly mutually exclusive characteristics. Practically speaking, it has been demonstrated that HSVR outperformed a number of ML-based models, namely artificial neural network (ANN), genetic algorithm (GA), and SVM [85].

The detail of HSVR has been mentioned elsewhere [83]. Briefly, a panel of SVR models was built by the *LIBSVM* package (software available at http://www.csie.ntu.edu.tw/~cjlin/libsvm) based on various descriptor combinations, and each SVR model represented a local model. The model generation and verification were executed using the modules *svm-train* and *svm-predict*, respectively, implemented in the *LIBSVM* package. The regression modes, namely, *ε*-SVR and *γ*-SVR, were adopted, and radial basis function (RBF) was employed as the kernel due to its simplicity and better performance when compared with the others [142]. The runtime parameters, namely regression modes *ε*-SVR and *ν*-SVR, the associated *ε* and *ν*, cost *C*, and the kernel width *γ*, were scanned by the systemic grid search algorithm using an in-house Perl script [143], in which all parameters were changed independently in a parallel fashion.

Two SVR models were initially adopted to develop an SVR ensemble (SVRE), which, in turn, was further subjected to regression by another SVR to yield the final HSVR model. The two-member SVREs were continuously assembled until the HSVR model performed well. Otherwise, the three- or even four-member ensembles were built by adding one or more SVR models, respectively, if all two-member ensembles failed to perform well. The descriptor selection and ensemble assembly were predominantly governed by the principle of Occam′s razor [144] by adopting the fewest descriptors and SVR models.

### 4.5. Predictive Evaluation

The predictivity of a generated model was evaluated by several statistic metrics. The coefficients *r*^2^ and *q*^2^ in the training set and external set, respectively, for the linear least square regression were computed by the following equation

(4) $$r^{2},\;q^{2}=1-\sum_{i=1}^{n}{\left({\hat{y}}_{i}-y_{i}\right)}^{2}/\sum_{i=1}^{n}{\left(y_{i}-\langle \hat{y}\rangle \right)}^{2}$$

where ${\hat{y}}_{i}$ and $y_{i}$ are the predicted and observed values, respectively; and $\langle \hat{y}\rangle$ and *n* stand for the average predicted value and the number of samples in the dataset, respectively.

Furthermore, the residual Δ*_i_*, which is the difference between $y_{i}$ and ${\hat{y}}_{i}$, was calculated

(5) $$\Delta_{i}=y_{i}-{\hat{y}}_{i}$$

The root mean square error (RMSE) and the mean absolute error (MAE) for *n* samples in the dataset were computed

(6) $$\mathrm{RMSE}={\left[\sum_{i=1}^{n}\Delta_{i}^{2}/n\right]}^{1/2},$$

(7) $$\mathrm{MAE}=\frac{1}{n}\sum_{i=1}^{n}\left|\Delta_{i}\right|,$$

The produced model was further subjected to 10-fold cross-validation instead of the widely used leave-one-out due to its better performance [145], giving rise to the correlation coefficient of 10-fold cross validation $q_{\mathrm{CV}}^{2}$. In addition to cross-validation, the developed models were also internally validated by the *Y*-scrambling test [102], which was carried out by randomly permuting the log ER values, viz. *Y* values, to refit the previously developed models while the descriptors were remained unaltered, giving rise to the correlation coefficient $r_{s}^{2}$. The observed log ER values were scrambled 25 times as suggested [107] to produce the average correlation coefficient $\langle r_{s}^{2}\rangle$. Furthermore, various modified versions of *r*^2^ proposed by Ojha et al. [110] were also computed

(8) $$r_{m}^{2}=r^{2}\left(1-\sqrt{\left|r^{2}-r_{o}^{2}\right|}\right),$$

(9) $${r^{\prime }}_{m}^{2}=r^{2}\left(1-\sqrt{\left|r^{2}-{r^{\prime }}_{o}^{2}\right|}\right),$$

(10) $$\langle r_{m}^{2}\rangle =\left(r_{m}^{2}+{r^{\prime }}_{m}^{2}\right)/2,$$

(11) $$\Delta r_{m}^{2}=\left|r_{m}^{2}-{r^{\prime }}_{m}^{2}\right|,$$

where the correlation coefficient $r_{o}^{2}$ and the slope of the regression line *k* were calculated from the regression line (predicted vs. observed values) through the origin, whereas $r_{o}^{\prime 2}$ was calculated from the regression line (observed vs. predicted values) through the origin.

Moreover, the correlation coefficients $q_{F1}^{2}$, $q_{F2}^{2}$, and $q_{F3}^{2}$ and concordance correlation coefficient (*CCC*) proposed by Shi et al. [146], Schüürmann et al. [147], Consonni et al. [148], and Chirico and Gramatica [149] were also computed by *QSARINS* [150,151] to evaluate the model performance in the external dataset

(12) $$q_{F1}^{2}=1-\sum_{i=1}^{n_{\mathrm{EXT}}}{\left(y_{i}-{\hat{y}}_{i}\right)}^{2}/\sum_{i=1}^{n_{\mathrm{EXT}}}{\left(y_{i}-\langle y_{\mathrm{TR}}\rangle \right)}^{2},$$

(13) $$q_{F2}^{2}=1-\sum_{i=1}^{n_{\mathrm{EXT}}}{\left(y_{i}-{\hat{y}}_{i}\right)}^{2}/\sum_{i=1}^{n_{\mathrm{EXT}}}{\left(y_{i}-\langle y_{\mathrm{EXT}}\rangle \right)}^{2},$$

(14) $$q_{F3}^{2}=1-\left[\sum_{i=1}^{n_{\mathrm{EXT}}}{\left(y_{i}-{\hat{y}}_{i}\right)}^{2}/n_{\mathrm{EXT}}\right]/\left[\sum_{i=1}^{n_{\mathrm{TR}}}{\left(y_{i}-\langle y_{\mathrm{TR}}\rangle \right)}^{2}/n_{\mathrm{TR}}\right],$$

(15) $$CCC=\frac{2\sum_{i=1}^{n_{\mathrm{EXT}}}\left(y_{i}-\langle y_{\mathrm{EXT}}\rangle \right)\left({\hat{y}}_{i}-\langle {\hat{y}}_{\mathrm{EXT}}\rangle \right)}{\sum_{i=1}^{n_{\mathrm{EXT}}}{\left(y_{i}-\langle y_{\mathrm{EXT}}\rangle \right)}^{2}+{\left({\hat{y}}_{i}-\langle {\hat{y}}_{\mathrm{EXT}}\rangle \right)}^{2}+n_{\mathrm{EXT}}{\left(\langle y_{\mathrm{EXT}}\rangle -\langle {\hat{y}}_{\mathrm{EXT}}\rangle \right)}^{2}}$$

where *n*_TR_ and *n*_EXT_ are the numbers of samples in the training set and external set, respectively; $\langle {\hat{y}}_{\mathrm{TR}}\rangle$ is the average predicted value in the training set; and $\langle y_{\mathrm{EXT}}\rangle$ and $\langle {\hat{y}}_{\mathrm{EXT}}\rangle$ are the average observed and predicted values in the external set, respectively.

Various criteria for those statistical parameters have been proposed to gauge the model predictivity [152]. For instance, Chirico and Gramatica considered that both $q_{F3}^{2}$ and *CCC* are the best validation parameters to measure the predictivity [149], whereas Roy et al. suggested that $\langle r_{m}^{2}\rangle$ and $\Delta r_{m}^{2}$ are the most stringent metrics [111]. Recently, Todeschini et al. demonstrated that $q_{F3}^{2}$ is the most reliable metric [112]. The parameter $q_{F2}^{2}$ has been adopted by Organization for Economic Co-operation and Development (OECD) to assess the performance of QSAR models [147].

More importantly, a model can be considered as predictive if it can meet the most stringent criteria collectively proposed by Golbraikh et al. [109], Ojha et al. [110], Roy et al. [111], and Chirico and Gramatica [112].

(16) $$r^{2},q_{\mathrm{CV}}^{2},\;q^{2},q_{\mathrm{Fn}}^{2}\geq 0.70,$$

(17) $$\left|r^{2}-q_{\mathrm{CV}}^{2}\right|<0.10,$$

(18) $$\left(r^{2}-r_{o}^{2}\right)/r^{2}<0.10\;\mathrm{and}\;0.85\leq k\leq 1.15,$$

(19) $$\left|r_{o}^{2}-{r^{\prime }}_{o}^{2}\right|<0.30,$$

(20) $$r_{m}^{2}\geq 0.65,$$

(21) $$\langle r_{m}^{2}\rangle \geq 0.65\;\mathrm{and}\;\Delta r_{m}^{2}<0.20,$$

(22) CCC≥0.85,

where *r* in Equations (18)–(21) represents the parameters *r* and *q* in the training set and external set, respectively; and *q*_Fn_ stands for *q*_F1_, *q*_F2_, and *q*_F3_.

## 5. Conclusions

P-gp substrate efflux can be a major obstacle in the success of CNS-targeted therapeutic delivery as well as a critical pharmacokinetic factor for causing DDIs. On the other hand, the CNS-related side-effects of non-CNS drugs can be reduced by P-gp mediated efflux. As such, P-gp substrate efflux is of critical importance to drug discovery and development regardless of CNS drugs or non-CNS drugs. An in silico model to predict the P-gp substrate efflux can be valuable to drug discovery and development. Nevertheless, P-gp substrate efflux is a complex process that can take place through various routes, namely active transport and passive permeability, leading to different descriptor combinations as well as different relationships to render these variations in different mechanisms. In this study, a QSAR predictive model derived from the novel hierarchical support vector regression (HSVR) scheme, which can simultaneously possess the advantageous characteristics of a local model and a global model, viz. broader coverage of applicability domain and higher level of predictivity, respectively, was developed to envisage the P-gp substrate efflux ratio. The developed HSVR showed great prediction accuracy for the 50 and 13 molecules in the training set and test set, respectively, with excellent predictivity and statistical significance. When mock tested by a group of molecules to mimic real challenges, the derived HSVR model also executed accordantly well. Furthermore, the HSVR model can elucidate the discrepancies among all published P-gp substrate classifiers, indicating its superiority. Hence, it can be affirmed that this HSVR model can be adopted as an accurate and reliable predictive tool, even in the high throughput fashion, to facilitate drug discovery and development by designing drug candidates with a more desirable pharmacokinetic profile.

## Figures and Tables

**Table 1:** Descriptor selected as the input of SVR models in the ensemble and their description.

Descriptor	SVR A	SVR B	SVR C	Description
SA			x^†^	Total surface area
*n*_N+O_	x	x		Number of nitrogen and oxygen atoms
*V*_m_	x	x	x	Molecule volume
PSA	x	x		Polar surface area
HBD	x	x		Number of hydrogen bond donating groups
*n*_Rot_		x	x	Number of rotatable bonds
*n*_Ar_		x		Number of aromatic rings

^†^ Selected.

**Table 2:** Statistic evaluations, namely correlation coefficient (*r*^2^), maximum residual (Δ_Max_), mean absolute error (MAE), standard deviation (*s*), RMSE, and 10-fold cross-validation correlation coefficient ($q_{\mathrm{CV}}^{2}$ ) evaluated by SVR A, SVR B, SVR C, and HSVR in the training set.

	SVR A	SVR B	SVR C	HSVR
*r* ^2^	0.95	0.95	0.98	0.96
Δ_Max_	0.32	0.51	0.40	0.45
MAE	0.11	0.07	0.02	0.06
*s*	0.12	0.10	0.06	0.10
RMSE	0.12	0.10	0.06	0.10
$q_{\mathrm{CV}}^{2}$	0.01	0.01	0.07	0.94

**Table 3:** Statistic evaluations, correlation coefficients *q*^2^, $q_{F1}^{2}$, $q_{F2}^{2}$, and $q_{F3}^{2}$, concordance correlation coefficient (*CCC*), maximal absolute residual (Δ_Max_), mean absolute error (MAE), standard deviation (*s*), and RMSE evaluated by SVR A, SVR B, SVR C, and HSVR in the test set.

	SVR A	SVR B	SVR C	HSVR
*q* ^2^	0.54	0.75	0.60	0.83
$q_{F1}^{2}$	0.39	0.67	0.55	0.80
$q_{F2}^{2}$	0.39	0.67	0.54	0.80
$q_{F3}^{2}$	0.38	0.66	0.54	0.80
*CCC*	0.45	0.86	0.78	0.87
Δ_Max_	0.60	0.42	0.55	0.42
MAE	0.29	0.22	0.24	0.17
*s*	0.35	0.26	0.30	0.22
RMSE	0.34	0.25	0.29	0.21

**Table 4:** Validation verification of HSVR based on prediction performance of those molecules in the training set and test set.

	Training Set	Test Set
*n*	50	13
$r_{o}^{2}$	0.95	0.77
*k*	1.03	1.05
${r^{\prime }}_{o}^{2}$	0.94	0.52
$r_{m}^{2}$	0.90	0.72
${r^{\prime }}_{m}^{2}$	0.85	0.60
$\langle r_{m}^{2}\rangle$	0.88	0.66
$\Delta r_{m}^{2}$	0.05	0.12
$r^{2},q_{\mathrm{CV}}^{2},\;q^{2},q_{\mathrm{Fn}}^{2}\geq 0.70$	x	x
$\left|r^{2}-q_{\mathrm{CV}}^{2}\right|<0.10$	x	N/A
$\left(r^{2}-r_{o}^{2}\right)/r^{2}<0.10\;\mathrm{and}\;0.85\leq k\leq 1.15$	x	x
$\left|r_{o}^{2}-{r^{\prime }}_{o}^{2}\right|<0.30$	x	x
$r_{m}^{2}\geq 0.65$	x	x
$\langle r_{m}^{2}\rangle \geq 0.65\;\mathrm{and}\;\Delta r_{m}^{2}<0.20$	x	x
*CCC* ≥ 0.85	N/A ^†^	x

^†^ Not applicable.

## Supplementary Materials

Table S1. Selected compounds for this study, their names, SMILES strings, CAS numbers, observed log ER values and predicted values by SVR A, SVR B, and HSVR, data partitions, and references; Table S2. Optimal runtime parameters for the SVR models; Figure S1. Molecular distribution for the samples in the training set (solid circle) and test set (open square) in the chemical space spanned by three principal components; Figure S2. Histograms of: (A) observed log ER; (B) molecular weight (MW); (C) polar surface area (PSA); (D) number of hydrogen bond acceptor (HBA); and (E) number of hydrogen bond donor (HBD) in density form for all molecules in the training set and test set.
