# Supplementary material for: Theoretical Prediction of the Complex P-Glycoprotein Substrate Efflux Based on the Novel Hierarchical Support Vector Regression Scheme
Source: Molecules. 2018 Jul 22;23(7):1820. doi: 10.3390/molecules23071820 (PMC6100076; doi:10.3390/molecules23071820)
Supplement: Supplementary file 1 [file molecules-23-01820-s001.zip › molecules-332763-supplementary figures.pdf]

Theoretical Prediction of the Complex P-Glycoprotein Substrate Efflux Based on the Novel Hierarchical Support Vector Regression Scheme

Chun Chen[1], Ming-Han Lee[1], Ching-Feng Weng[2], and Max K. Leong[1,2,*]

[1]Department of Chemistry, National Dong Hwa University, Shoufeng, Hualien 97401, Taiwan; 610212011@ems.ndhu.edu.tw (C.C.); 610512018@gms.ndhu.edu.tw (M-H.L.)

[2]Department of Life Science and Institute of Biotechnology, National Dong Hwa University, Shoufeng, Hualien 97401, Taiwan; cfweng@gms.ndhu.edu.tw

[*]Correspondence: leong@gms.ndhu.edu.tw; Tel: +886.3.890.3609

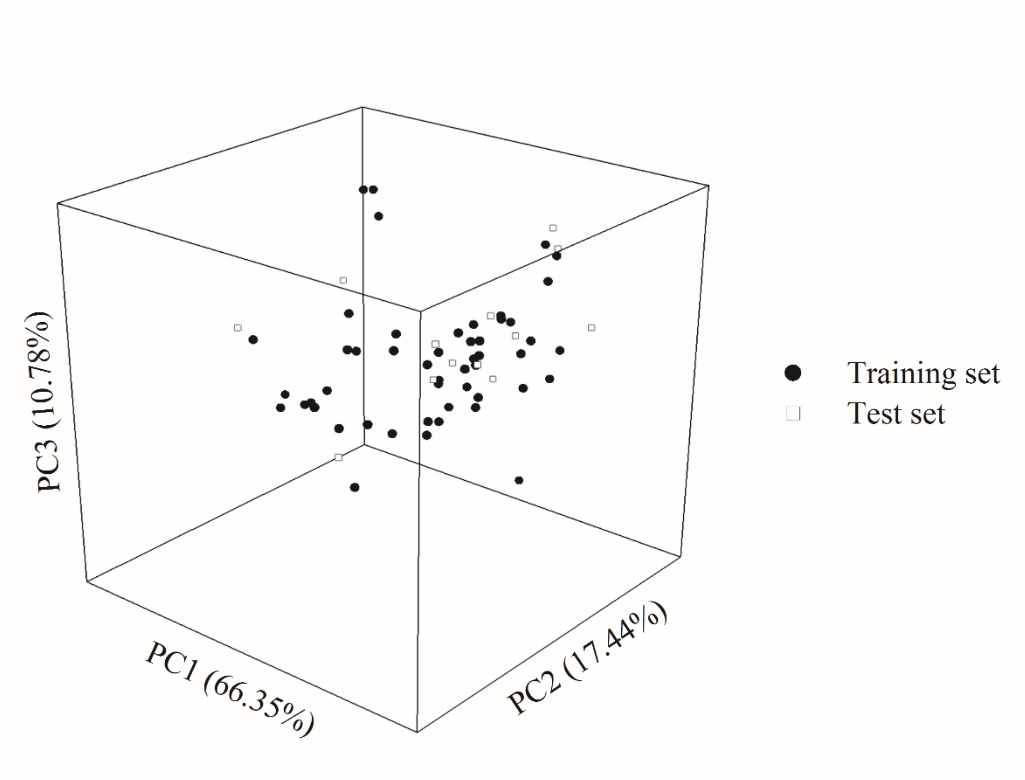

Figure S1. Molecular distribution for the samples in the training set (solid circle) and test set (open square) in the chemical space spanned by three principal components.

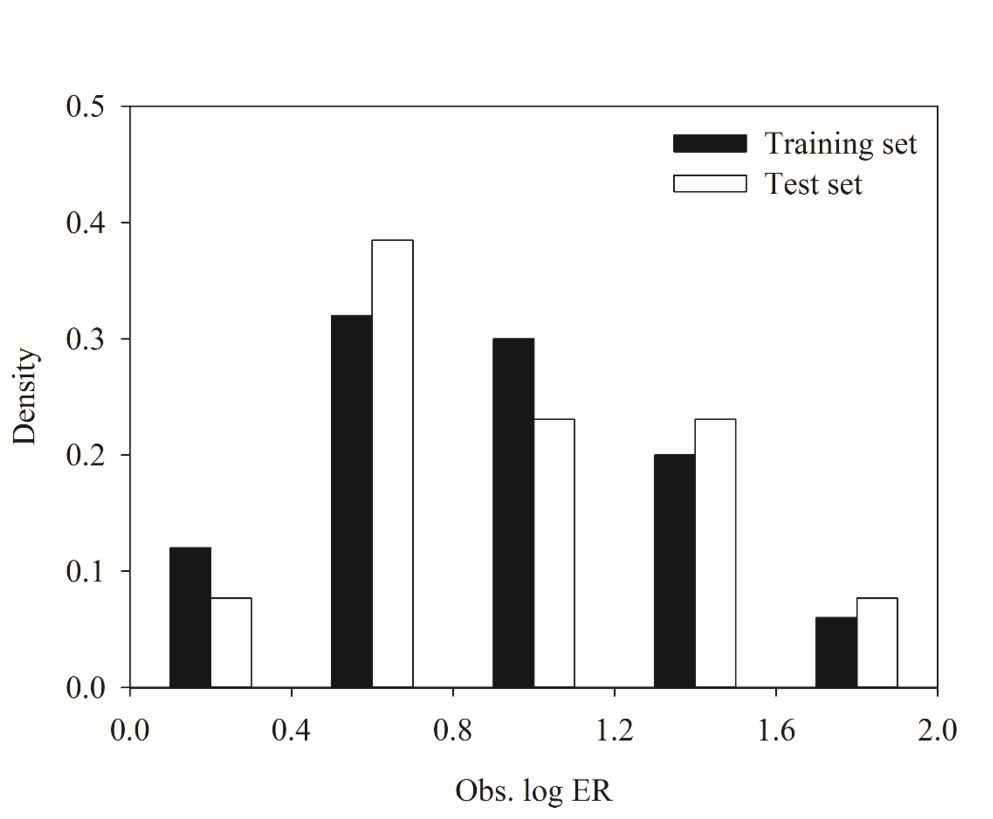
Training set
Test set
Density
Obs. log ER
0.0
0.1
0.2
0.3
0.4
0.5
0.0
0.4
0.8
1.2
1.6
2.0

(A)

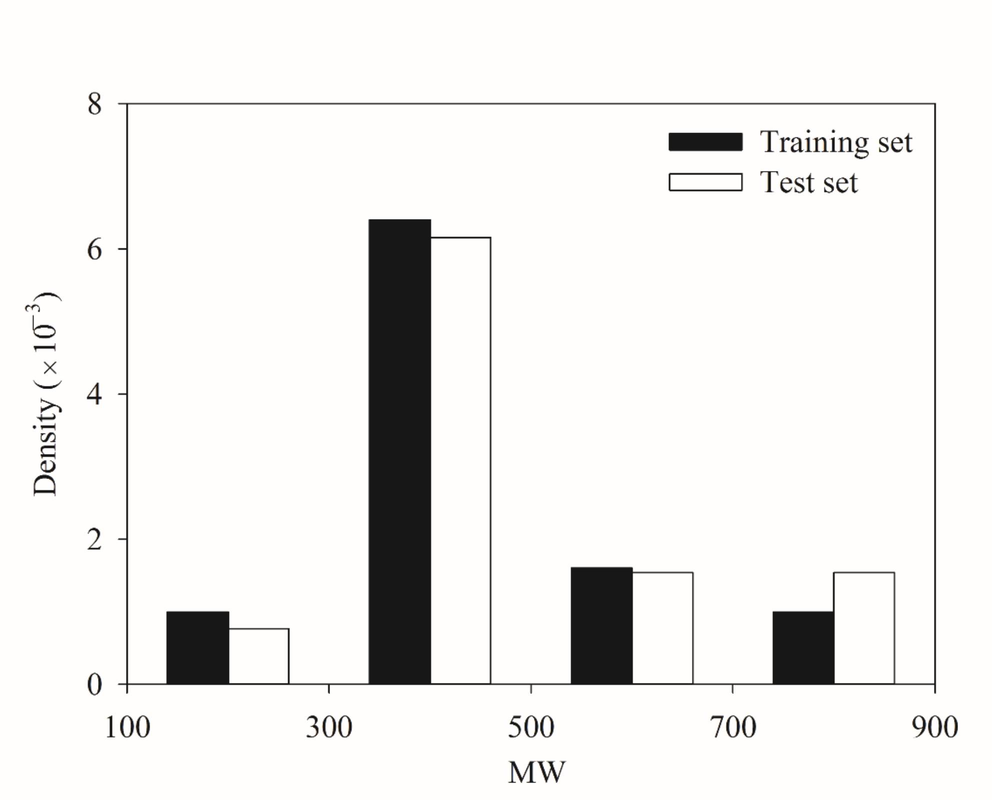
Training set
Test set
Density ($\times 10^{-3}$)
MW
0
2
4
6
8
100
300
500
700
900

(B)

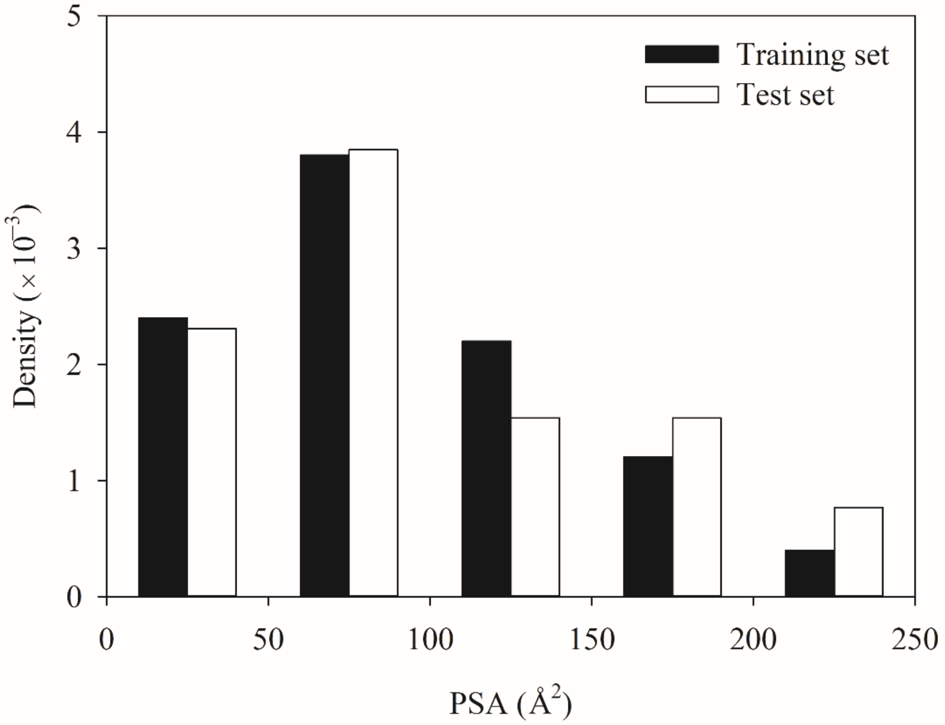
Training set
Test set
Density (× 10⁻³)
PSA (Å²)
0
1
2
3
4
5
0
50
100
150
200
250

(C)

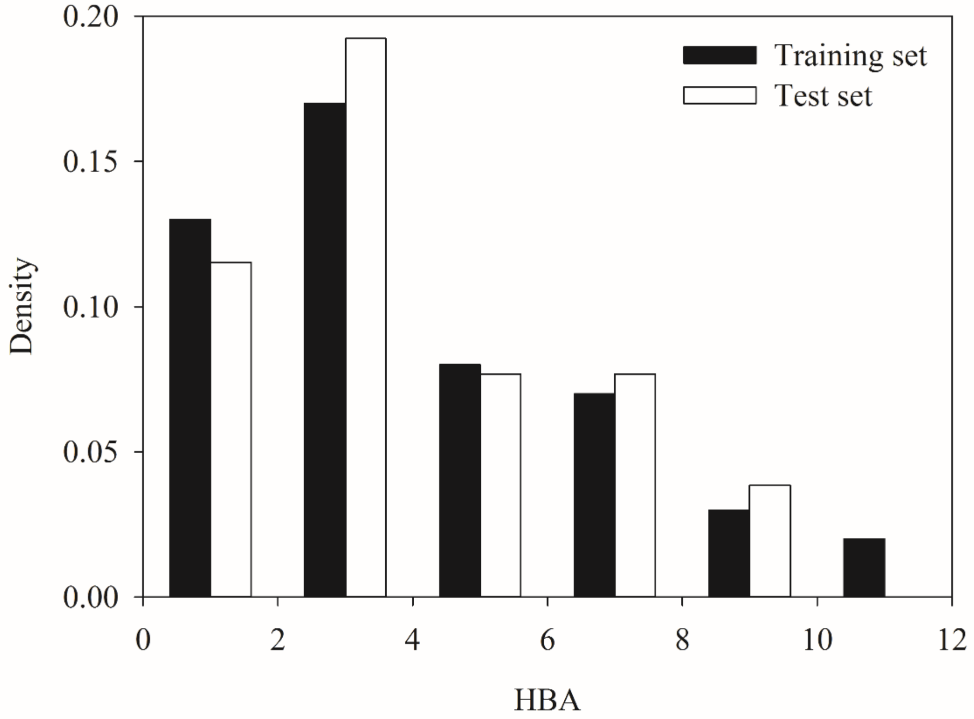
Training set
Test set
Density
HBA
0.00
0.05
0.10
0.15
0.20
0
2
4
6
8
10
12

(D)

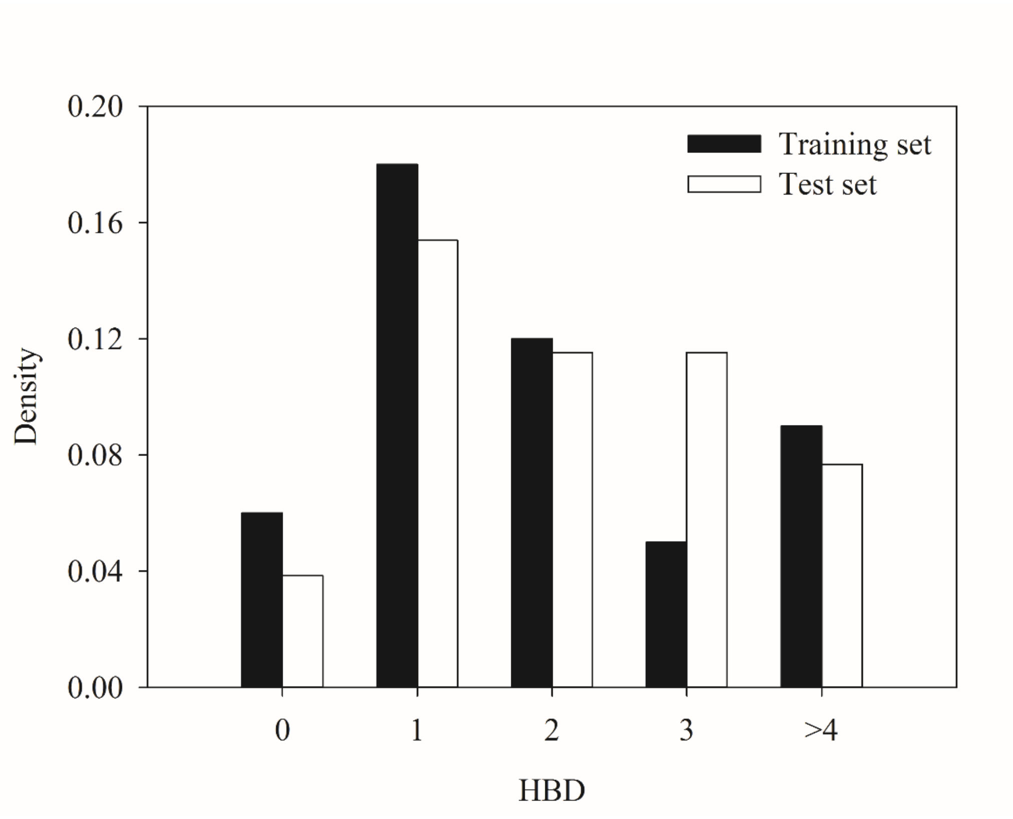

(E)

Figure S2. Histograms of (A) observed log ER, (B) molecular weight (MW), (C) polar surface area (PSA), (D) number of hydrogen bond acceptor (HBA), and (E) number of hydrogen bond donor (HBD) in density form for all molecules in the training set and test set.
